# How healthcare structures and communication delivery influence trust: a parallel-group randomized controlled trial

**DOI:** 10.1007/s10389-021-01620-z

**Published:** 2021-07-12

**Authors:** Stephen Cantarutti, Emmanuel M. Pothos

**Affiliations:** Department of Psychology, City, University of London, Northampton Square, London, EC1V 0HB UK

**Keywords:** Determinants of trust, Healthcare, Dimensions of trust

## Abstract

**Background:**

According to recent polling, public trust in the healthcare sector remains low relative to other industries globally. The implications of low healthcare trust permeate throughout the industry in a number of ways, most visibly by discouraging therapy compliance.

**Methods:**

This study investigated four putative determinants of trust in healthcare-related scenarios: individuals vs. collective groups as communicators of healthcare advice; expert vs. laypeople as providers of healthcare communication; public vs. private healthcare sector; and positive vs. negative information. Two hundred seventy-four participants were recruited via Prolific Academic and were presented with four statements in random order, related to a positive reflection of the public healthcare sector, a negative reflection of the public healthcare sector, a positive reflection of the private healthcare sector and a negative reflection of the private healthcare sector. According to these reflection, participants were repeatedly asked to rate the system on its trustworthiness. Trust outcomes were constructed using a four-dimension framework, consisting of benevolence, reliability, competence and predictability.

**Results:**

Claims relating to the public sector had a significantly stronger impact on benevolence and reliability than claims relating to the private sector; claims from individuals had a significantly stronger impact on all trust variables than claims from collectives; and claims from laypeople had a significantly greater impact on reliability and competence ratings than claims from experts.

**Conclusions:**

The findings in this study offer insight into the patterns with which trust decisions are made in healthcare contexts. More importantly, this research offers a novel perspective of how different factors interact to affect the various facets of trust. These results provide a foundation for future study in this evolving area, and offer insights into designing effective communication strategies that cultivate greater levels of individual trust in the healthcare sector.

**Supplementary Information:**

The online version contains supplementary material available at 10.1007/s10389-021-01620-z.

## Background

In times of health emergencies, it is imperative that people adhere to guidelines issued by national healthcare providers and governments. In the case of a pandemic outbreak, low levels of compliance with established healthcare standards risks greater disease burden and rates of transmission (Koo et al. 2020; Lewnard and Lo 2020). However, adherence to restrictions must partly originate from *trust* in these guidelines. Accordingly, understanding the determinants of trust judgments is crucial in communicating trust-fostering healthcare guidelines to a population.

The development of trust is complex, both because there are multiple putative determinants of trust, and because trust itself is a multifaceted concept. Existing work in healthcare provision has tended to focus on individual determinants of trust, in isolation from one another. For example, patients’ willingness to trust differs significantly across public vs. private healthcare providers (Calnan and Sanford 2004; Meyer 2015; Ozawa and Walker 2011). Additionally, perhaps unsurprisingly, people are more likely to accept healthcare advice from expert sources, compared to novice sources (Van Swol and Sniezek 2005; Yaniv and Kleinberger 2000). Moreover, research on the importance of intimacy in trust relationships sheds light on how people are more likely to offer trust in one-on-one relationships, relative to groups (McEvily et al. 2002; Tabrani et al. 2018). Finally, research on the impact of framing effects on trust suggests negative messages are more powerfully received than positive messages because they are more trusted (Cobb 2005).

Notwithstanding the value of these findings, understanding the way trust develops in realistic (e.g. healthcare) communications requires an appreciation of how these putative determinants potentially offset or strengthen one another. A major objective of the present work is to bring these factors together and study their interactions’ impact on trust.

It is well established that trust is a multi-faceted concept (Zaheer et al. 1998; Aljukhadar et al. 2010). Our decision to build a unique four-pillared trust framework, consisting of benevolence, reliability, competence and predictability, draws from seminal interdisciplinary abstract conceptualizations of trust to account for this (Baier 1986; Lewis and Weigert 1985; Newton and Norris 1999). Brown et al.’s (2011) definition of healthcare trust captures the four pillars well: ‘(trust is) expectations which relate to the ability and willingness of the trustee to affect certain outcomes. Trust is therefore dependent on the professional’s ability to demonstrate that their actions are embedded within norms of competency and care’. From this definition, we can extract each of the four pillars presented in the framework: benevolence is visible in the *willingness* of the carer and the general norms of care; reliability and predictability are present in the expectations that the carer will affect certain outcomes; and competence is seen in the carer’s *ability* to affect outcomes. In the present study, we invited participants to reflect on trust-related factors, to determine precisely how each factor may affect the different dimensions of trust.

To dissociate prior trust attitudes from attitudes solely arising from experimental manipulations, we opted to introduce hypothetical healthcare situations. Participants were asked to provide trust evaluations on the four pillars of trust, in a within-participants design. Benevolence was defined as equality amongst patients, accessible treatment and honesty from healthcare providers. Reliability was defined as responsible resource allocation, safety and accessible treatment. Competence was defined as broader system quality and individual technical competence. Finally, predictability was defined as consistency in the quality of healthcare provided.

Each variable was evaluated on a linear scale, with 1 and 5 anchors corresponding to strongly agree and strongly disagree, respectively. Overall, each participant provided four (statements) x four (trust variables) ratings for a total of 16 ratings.

## Methods

Participants were recruited via Prolific Academic (*N* = 275; mean age = 30.67, age range = 18–82) to complete an original questionnaire, developed for the purposes of this study; 129 participants identified as male, while 146 participants identified as female. Sample size was determined a priori from practical considerations, lacking prior work to offer a more concrete guide. Inclusion criteria stated that participants must have been at least 18 years of age at the time of survey completion, and fluent in English. Questionnaire response times (M = 588 s, SD = 274 s) 3 SDs above or below the mean for overall study completion were excluded. Three participants were excluded from the analysis for failing to complete the survey in less than 26 min, the upper time limit, according to our exclusion criteria.

Participants were asked to imagine themselves as a citizen experiencing health problems in a hypothetical country, Country Beta. They were asked to evaluate four relevant statements regarding healthcare provided by different sources within that hypothetical country. Participants were provided with a contextual description prior to answering questions (see Appendix A).

After reading these initial instructions and assuming the role of this imagined patient, participants were confronted with four sets of four questions. In each set, participants were initially presented with a recommendation, reflection or piece of information from one of four sources: themselves, written as *you* in the experiment; the surgeon general of Country Beta; a group of friends familiar with the imagined health situation; and a group of surgeons from Country Beta.

Along with the four possible sources of information (you, the surgeon general, a group of friends, or a group of surgeons) which capture our expert vs. lay and individual vs. collective communication factors (for example, the surgeon general would represent the expert and individual factor categories), we manipulated whether the healthcare statement concerned the public or private sector and varied whether the health statements were positive or negative. These four factors together represented our determinants of trust factors.

Within participants, we crossed each of the factors, presenting participants with 16 statements in total (see Appendix B), in a 2 (positive vs. negative) × 2 (private vs. public) × 2 (collective vs. individual) × 2 (expert vs. lay) design. In total, participants would therefore encounter: a positive reflection of the public healthcare sector, a negative reflection of the public healthcare sector, a positive reflection of the private healthcare sector, and finally, a negative reflection of the private healthcare sector. Participants were presented with the statements in random order, both in terms of content (private/public and positive/negative) and in terms of communicator (individual/collective and lay/expert). Note, each participant would receive non-repetitive information: individual participants would receive information from each of the four sources only once, with each piece of information relating to a different scenario, to ensure that all participants were exposed to information from each of the four possible sources.

Based on their interpretation of each statement, participants were asked to evaluate Country Beta’s healthcare system as a whole (that is, not focused on the specific information they received, but rather on the system’s trustworthiness, as they perceived it, based on the information they received) on four trust-related variables: benevolence, reliability, competence and predictability. The statements were designed to allow participants to form an impression regarding their trust for the healthcare system as a whole. The degree to which different factors presented within the statements influenced participants’ personal assessment of the healthcare system was the primary interest of this study.

Taken together, the sources of information, the positive/negative valence of the information, and the relevant healthcare sector captured the four putative determinants of trust we studied in this experiment: individual/collective source of information; expert/lay source of information; positive/negative information; and public/private healthcare sector. The statistical impact of each individual factor on trust ratings was determined using separate linear mixed-effects models on each of the four trust-related variables.

This is an example of a typical scenario: *In a previous visit to a private clinic in Country Beta, stemming from similar problems with dizziness,* ***you*** *received very high-quality service, offering a planned sleep schedule that helped temporarily eliminate your symptoms. As such,* ***you*** *are inclined to visit your private clinic to get an assessment. Please now provide your impression of Country Beta’s healthcare system by answering the following four questions* (Appendices A and B offer the full relevant text)*.*

This particular example references ‘you’ as the source of information, in the context of a positive private healthcare sector scenario. Upon reading this description, participants would be asked to evaluate Country Beta’s healthcare system on its benevolence, reliability, competence and predictability; each variable was briefly defined after each statement to avoid interpretive ambiguity on the part of participants.

Participant condition allocations were randomized and counterbalanced to ensure that participants had equal exposure to positive and negative impressions of both the public and private sectors, from each of the sources presented. Scores of the negative impression statements were reversed to ensure that, in all cases, higher ratings could be interpreted as statements having a greater impact on trust variables. (See Appendix B for a full list of statements provided to participants.)

## Results

We conducted a series of linear mixed effects model analyses for each of the four dependent variables studied in this experiment: benevolence, reliability, competence and predictability. To identify the best model, we employed a series of hierarchically related models. In all models ‘participants’ served as the random effect, while public vs. private, positive vs. negative, lay vs. expert, and individual vs. collective served as fixed effects independent variables. In simpler models, random effects were modelled with intercepts, while slopes were included for fixed effects only; in the more complex models, random effects were modelled with intercepts and slopes. Models were optimized in terms of maximum likelihood with the resulting minus two log likelihoods (−2LL) used to evaluate nested models using chi-squared tests, on degrees of freedom corresponding to differences in model parameters. We selected the most complex model that offered a significant improvement relative to the immediately less complex corresponding model. Upon running the same procedure across each of our dependent variables, the best model for each dependent variable was the one for which random effects were modelled with both intercepts and slopes (here and elsewhere, for all main fixed effects), with a variance components covariance matrix, and two-way interactions for all fixed effects. This procedure was repeated for each of the four dependent variables (benevolence, reliability, competence and predictability). (Full results are presented in Appendix C.)

### Benevolence results

The dependent variable in this analysis was participants’ benevolence rating of the healthcare system. For the best model, as above, we observed a significant main effect for the positive vs. negative independent variable, the public vs. private variable, the individual vs. collective variable, and the expert vs. lay*public vs. private interaction. Mean comparisons (see Appendix D) indicated that positive statements were significantly more impactful than negative statements in generating high benevolence ratings; that statements pertaining to the public healthcare sector were significantly more impactful than statements related to the private healthcare sector; and that statements from individuals were significantly more impactful than statements from collectives. Further investigation into the significant interaction terms revealed that participants’ benevolence ratings were more impacted by expert statements relating to the public sector than statements from laypeople, while statements from laypeople relating to the private sector had greater impact on benevolence ratings than statements from experts.

### Reliability results

Reliability served as the dependent variable in our second analysis. We observed a significant main effect for the positive vs. negative variable, the public vs. private variable, the expert vs. lay variable, the individual vs. collective variable, the public vs. private*expert vs. lay interaction, and public vs. private*individual vs. collective interaction. Further analysis of the individual categories within these variables provided greater insights into the nature of their respective relationships with the reliability measure. Positive statements were significantly more impactful than negative statements in influencing reliability ratings; statements relating to the public healthcare sector were significantly more impactful than statements relating to the private sector; and the same holds true for statements provided by laypeople compared to statements from experts and for statements communicated by individuals as opposed to collectives. Additional analysis of the public vs. private*expert vs. lay interaction suggested that statements related to the public sector were more impactful on reliability ratings when delivered by experts than laypeople, while statements related to the private sector had a greater impact on reliability ratings when delivered by laypeople than by experts. Finally, statements related to the public sector had a greater impact on reliability ratings when delivered by individuals than by collectives, while statements related to the private sector were only marginally more impactful on reliability ratings when delivered by individuals than by collectives.

### Competence results

Competence served as the dependent variable in this analysis. We observed a significant main effect for the positive vs. negative variable, the expert vs. lay variable, the individual vs. collective variable, the positive vs. negative*public vs. private interaction and the public vs. private*expert vs. lay interaction. Positive statements were significantly more impactful in informing competence ratings than negative statements, while statements from laypeople had a greater impact on competence ratings than statements from experts. Additionally, statements from individuals had a greater impact on competence ratings than statements from collectives. Examination of the interaction terms revealed that positive statements had a greater impact on competence ratings when referring to the public healthcare sector than the private sector, while negative statements had a greater impact on competence when referring to the private sector than the public sector. Additionally, statements from experts registered a greater impact on competence ratings when referring to the public sector than statements from laypeople, while statements from laypeople were more impactful in terms of competence ratings when referring to the private system than statements from experts.

### Predictability results

With our predictability measure serving as the dependent variable in this analysis, we observed a significant main effect for the positive vs. negative variable, the individual vs. collective variable and the public vs. private*expert vs. lay interaction. Further analysis of the relationships between these variables and our dependent variable, predictability, revealed that participants were more impacted in terms of their predictability ratings by positive statements than negative statements, and by statements from individuals than statements from collectives. Additionally, mean comparisons conducted on the critical interaction variable, public vs. private*expert vs. lay, revealed that predictability ratings were more impacted by expert statements when referring to the public sector than laypeople statements, but that statements from laypeople had a greater impact on predictability ratings in relation to the private sector than statements from experts.

## Discussion

Our intention in this study was to explore four putative determinants of trust in healthcare, based on impressions created by participants after encountering specific statements and pieces of information concerning a fictional healthcare system.

We observed a series of surprising results. Across each of the four trust-related dependent variables, positive statements impacted trust more so than their matched negative statements, a finding analogous to reports that positive information may impact belief updating more strongly (Sharot et al. 2012). Healthcare systems also attracted higher trust ratings for reliability, predictability and competence, following statements from individuals, as opposed to collectives. This is perhaps because communications from individuals presented less opportunity for dissent and contradiction. Theoretically, this appears to corroborate research suggesting that offering a more salient, implementable, simple direction for participants to follow may cultivate greater feelings of trust, as evidenced by recent developmental studies (Einav 2018; Guerrero et al. 2016). Practically, this presents relevant insights to healthcare messaging efforts directed towards the public. In light of the COVID-19 pandemic, for example, presenting messages as communicated by a single, salient individual, as opposed to a broader, more anonymous collective, may foster greater trust and greater adherence to messages.

Contrary to our initial expectations, statements from lay sources resulted in stronger shifts in both reliability and competence ratings. Studies evaluating healthcare workers’ motivations have revealed that ‘goal displacement’, the phenomenon wherein ‘rule following becomes a means to an end other than that intended by the designers of the system’, (Marshall and Harrison 2005) is a genuine consequence of changes in incentive structure. In the present context, rather than showing motivation towards the goal of offering high quality patient care, participants may have considered experts’ goals to be misdirected, leading to lower expert trust ratings. Theoretically, this finding provides a counterargument to the reputation heuristic, which suggested that people would be more willing to accept advice from expert, rather than novice, advisors (Yaniv and Kleinberger 2000). Practically, this suggests that clearer transparency practices from experts, with respect to compensation primarily, may help to re-establish the honesty of their intentions and work practices, and (appropriately) rehabilitate trust in experts, as a result.

Additionally, it is impossible to eschew pre-existing biases towards healthcare entirely. Public healthcare was likely viewed as significantly more benevolent and reliable, perhaps on the grounds that public healthcare is frequently seen as more accessible than exclusive private healthcare offerings, particularly in the UK, where the majority of our participants were located (Calnan and Sanford 2004). Practically, utilizing this apparent existing groundswell of goodwill towards the NHS appears to be an effective way of fostering trust. Identifying the NHS as a unified, individual entity in public messaging could effectively leverage citizens’ tendency to trust both the public sector and individual messengers.

Finally, we discovered that all trust ratings were significantly more impacted by expert claims in the context of the public sector, but significantly more impacted by laypeople claims in the context of the private sector. As the vast majority of expert opinion in the UK, related to healthcare, comes from the public sector, it is unsurprising that expert opinion would be more impactful in public sector scenarios, while lay perspectives would, conversely, be more impactful in private sector contexts. Theoretically, this further develops our understanding of how multiple factors (healthcare sector and expert/lay communicators) affecting trust interact to determine trust ratings, offering a more nuanced view of the underpinnings of trust, while better replicating environmental contexts.

## Conclusions

Several potential avenues for extending the present work remain. Notably, researchers can consider whether is it valid to assume that trust ratings from limited information reliably translate to impressions about an entire healthcare system; researchers can ask whether pre-existing biases about trust in healthcare and the present manipulations can be separated; and questions of whether the ‘fast’ (Kahneman 2011) evocation of trust judgments in brief experimental contexts is characteristic of trust attitudes in general can be addressed. Nevertheless, we contend that trust evaluations in real life generally involve non-elaborative impressions from partial information (Beldad et al. 2010; Lewis and Weigert 1985). As a result, our paradigm is arguably a reasonable approximation for how trust develops in reality.

In closing, this study offers potentially valuable information to governing bodies because it aims to capture trust in a realistic decision-making context. By developing the four-pillared framework for trust and exploring how trust-related factors interact, we believe we come closer to portraying realistic trust-related decision-making. Accordingly, as governments worldwide strive to improve trust via healthcare guidelines, we hope that these findings offer valuable insight into designing guidelines that better foster trust.

## Supplementary Information

ESM 1 (DOCX 20 kb)

## Data Availability

All data generated or analysed during this study are included in this published article [and its supplementary information files].

## Code Availability

All data generated or analysed during this study are included in this published article [and its supplementary information files].
