# Supplementary material for: How healthcare structures and communication delivery influence trust: a parallel-group randomized controlled trial
Source: Z Gesundh Wiss. 2021 Jul 12;31(6):831–6. doi: 10.1007/s10389-021-01620-z (PMC8274661; doi:10.1007/s10389-021-01620-z)
Supplement: Supplementary file 1 — (DOCX 20 kb) [file 10389_2021_1620_MOESM1_ESM.docx]

How Healthcare Structures and Communication Delivery Influence Trust: a Parallel-Group Randomized Controlled Trial – Supplementary Information

Appendix A—an example of the hypothetical healthcare description participants received.

Please imagine that you are a hypothetical resident of Country Beta. Country Beta has a dual-provider healthcare system, offering both private and public healthcare services. As a citizen of Country Beta with private health insurance, you have access to healthcare treatment from both the public and private sectors. You have been experiencing spells of dizziness over the last two weeks and are currently assessing your treatment options. You have chronically low blood pressure and at various points in your life have experienced light-headedness. Over the past two weeks, symptoms have worsened, and, feeling concerned, you are weighing your options in addressing the problem.  According to the information you have been provided about Country Beta's healthcare system, you will now be asked to give your impression of the nation's healthcare system.

Appendix B—the statements presented to participants

| Source of Information | Statement of Advice 1 | Statement of Advice 2 | Statement of Advice 3 | Statement of Advice 4 |
| --- | --- | --- | --- | --- |
| Personal Opinion | In a previous visit to a private clinic in Country Beta, stemming from similar problems with dizziness, you received very high-quality service, offering a planned sleep schedule that helped temporarily eliminate your symptoms. As such, you are inclined to visit your private clinic to get an assessment. | In a previous visit to a private clinic in Country Beta, stemming from similar problems with dizziness, you received very low-quality service, offering a planned sleep schedule that did not help temporarily eliminate your symptoms. As such, you are inclined to visit a public clinic to get an assessment. | Having experienced long waiting times to seeing a medical practitioner in the public healthcare system in the past, you are slightly apathetic to attending to the symptoms and feel inclined to wait an additional week, to see whether the dizziness passes. | Having read a very complimentary piece on the public system’s established course of action to individuals exhibiting your symptoms, you intend to get preliminary bloodwork done immediately. |
| Surgeon General | You read that the surgeon general of Country Beta spoke highly of the quality of care at private clinic services, in cases similar to yours. As such, he would recommend that you visit your private clinic to get an assessment. | You read that the surgeon general of Country Beta spoke poorly of the quality of care at private clinic services, in cases similar to yours. As such, he would recommend that you visit a public clinic to get an assessment. | Commenting on the public healthcare system’s long waiting times, the surgeon general in Country Beta has recommended that people wait an additional week to see whether the dizziness passes. | Complimenting the public system’s established course of action for individuals exhibiting your symptoms, the surgeon general of Country Beta would recommend that you get preliminary bloodwork done immediately. |
| Group of friends | In previous visits to private clinics in Country Beta, several friends have spoken highly of the quality of care received in cases similar to yours. As such, they recommend that you visit your private clinic to get an assessment. | In previous visits to private clinics in Country Beta, several friends have spoken poorly of the quality of care received in cases similar to yours. As such, they recommend that you visit your private clinic to get an assessment. | Several friends familiar with your symptoms in the past recommend that you wait an additional week to see whether the dizziness passes because of the public healthcare system’s long waiting times. | Having collectively read complimentary research of the public system’s established course of action for individuals exhibiting your symptoms, several friends recommend you get preliminary bloodwork done immediately. |
| Group of surgeons | You read that a group of surgeons in Country Beta spoke highly of the quality of care at private clinic service in cases similar to yours. As such, they recommend that you visit your private clinic to get an assessment. | You read that a group of surgeons in Country Beta spoke poorly of the quality of care at private clinic service in cases similar to yours. As such, they recommend that you visit a public clinic to get an assessment. | For people who experience similar symptoms, a group of surgeons in Country Beta recommend that people wait an additional week to see whether the dizziness passes because of the public healthcare system’s long waiting times. | Collectively complimenting the public system’s established course of action for individuals exhibiting your symptoms, a group of surgeons in Country Beta would recommend that you get preliminary bloodwork done immediately. |

Appendix C—detailed statistical results

*Mixed Effects Model Significant Results*

|  | Benevolence | Reliability | Competence | Predictability |
| --- | --- | --- | --- | --- |
| Positive/Negative (yellow = negative > positive; green = positive > negative) | (F(1, 542.10) = 119.05, p < .001), 95% CI [0.53, 0.76] | F(1, 543.24) = 62.44, p < .001, 95% CI [0.34, 0.57] | F(1, 544.39) = 110.29, p < .001, 95% CI [0.51, 0.74] | F(1, 544.78) = 62.09, p < .001, 95% CI [0.34, 0.57] |
| Public/Private (yellow = private > public; green = public > private) | (F(1, 542.10) = 25.17, p < .001), 95% CI [0.13, 0.30] | F(1, 543.25) = 17.25, p < .001, 95% CI [0.10, 0.28] |  |  |
| Expert/Lay (yellow = expert > lay; green = lay > expert) |  | F(1, 874.26) = 4.22, p = .04, 95% CI [0, 0.20] | F(1, 838.81) = 5.54, p = .019, 95% CI [0.02, 0.21] |  |
| Individual/Collective (yellow = individual > collective; green = collective > individual) | (F(1, 805.65) = 5.59, p = .018, 95% CI [-0.02, 0.20] | F(1, 852.80) = 5.17, p = .023 95% CI [0.02, 0.21] | F(1, 818.77) = 4.98, p = .026, 95% CI [0.01, 0.20] | F(1, 820.16) = 4.39, p = .037, 95% CI [0.01, 0.19] |
| Positive/Negative*Public/Private  (third cell: positive > negative in public; negative > positive in private) |  |  | F(1, 544.43) = 8.28, p = .004 |  |
| Public/Private*Expert/Lay  (all cells = public > private in expert; private > public in lay) | (F(1, 1091.98) = 29.19, p < .001) | F(1, 1081.37) = 36.36, p < .001 | F(1, 1091.11) = 48.94, p < .001 | F(1, 1090.96) = 18.62, p < .001 |
| Public/Private*Individual/Collective (second cell = public > private in individual; private > public in collective) |  | F(1, 1071.07) = 4.13, p = .042 |  |  |

Appendix D—detailed information for the significant effects

| Factor | Benevolence | Reliability | Competence | Predictability |
| --- | --- | --- | --- | --- |
| *Positive/Negative*  Positive  Negative | (M = 3.577, SE = .035)  (M = 2.934, SE = .04) | (M = 3.562, SE = .037)  (M = 3.110, SE = .039) | (M = 3.619, SE = .037)  (M = 2.996, SE = .04) | (M = 3.454, SE = .035)  (M = 3.002, SE = .038) |
| *Public/Private*  Public  Private | (M = 3.36, SE = .04, 95% CI [3.29, 3.43])  (M = 3.14, SE = .04, 95% CI [3.07, 3.22]) | (M = 3.43, SE = .04, 95% CI [3.36, 3.50])  (M = 3.24, SE = .04, 95% CI [3.16, 3.31]) |  |  |
| *Expert/Lay*  Expert  Lay |  | (M = 3.28, SE = .04, 95% CI [3.21, 3.36])  (M = 3.38, SE = .04, 95% CI [3.31, 3.46]) | (M = 3.25, SE = .04, 95% CI [3.17, 3.32]).  (M = 3.36, SE = .04, 95% CI [3.29, 3.44]) |  |
| *Individual/Collective*  Individual  Collective | (M = 3.31, SE = .04, 95% CI [3.23, 3.38])  (M = 3.20, SE = .04, 95% CI [3.12, 3.27]) | (M = 3.39, SE = .04, 95% CI [3.31, 3.46])  (M = 3.28, SE = .04, 95% CI [3.20, 3.35]). | (M = 3.36, SE = .04, 95% CI [3.28, 3.43])  (M = 3.25, SE = .04, 95% CI [3.18, 3.33]). | (M = 3.28, SE = .04, 95% CI [3.20, 3.35])  (M = 3.18, SE = .04, 95% CI [3.11, 3.25]) |
| *Expert/Lay*Public/Private*  Expert – Public  Lay – Public  Expert – Private  Lay - Private | (M = 3.46, SE = .05)  (M = 3.26, SE = .05)  (M = 3.33, SE = .05)  (M = 2.96, SE = .05) | (M = 3.54, SE = .05, 95% CI [3.44, 3.64])  (M = 3.32, SE = .05, 95% CI [3.22, 3.42]),  (M = 3.03, SE = .05, 95% CI [2.93, 3.13]).  (M = 3.45, SE = .05, 95% CI [3.35, 3.55]) | (M = 3.46, SE = .05, 95% CI [3.36, 3.56])  (M = 3.20, SE = .05, 95% CI [3.10, 3.30]),  (M = 3.04, SE = .05, 95% CI [2.94, 3.14]).  (M = 3.52, SE = .05, 95% CI [3.42, 3.62]) | (M = 3.34, SE = .05, 95% CI [3.25, 3.44])  (M = 3.16, SE = .05, 95% CI [3.06, 3.26])  (M = 3.08, SE = .05, 95% CI [2.98, 3.17])  (M = 3.33, SE = .05, 95% CI [3.23, 3.43]) |
